# Role of Hepatitis E Virus Infection in Acute-on-Chronic Liver Failure

**DOI:** 10.1155/2018/9098535

**Published:** 2018-06-28

**Authors:** Mario Frias, Pedro López-López, Antonio Rivero, Antonio Rivero-Juarez

**Affiliations:** Clinical Virology and Zooneses, Hospital Universitario Reina Sofía de Córdoba, Instituto Maimonides de Investigación Biomédica de Córdoba (IMIBIC), Universidad de Córdoba, Spain

## Abstract

Chronic liver disease (CLD) with a variety of causes is currently reported to be one of the main causes of death worldwide. Patients with CLD experience deteriorating liver function and fibrosis, progressing to cirrhosis, chronic hepatic decompensation (CHD), end-stage liver disease (ESLD), and death. Patients may develop acute-on-chronic liver failure (ACLF), typically related to a precipitating event and associated with increased mortality. The objective of this review was to analyze the role of acute hepatitis E virus (HEV) infection in patients with CLD, focusing on the impact of this infection on patient survival and prognosis in several world regions.

## 1. Introduction

Chronic liver disease (CLD) is a pathology characterized by continuous, progressive damage to the liver tissue [1]. Several conditions can cause this pathology, such as viral hepatitis (hepatitis C [HCV] and B [HBV]), metabolic disorders (Wilson's disease and nonalcoholic fatty liver), toxic or drug abuse (alcohol consumption), and autoimmune disorders [2]. Patients with CLD experience deterioration of liver function and fibrosis, progressing to cirrhosis, chronic hepatic decompensation (CHD), end-stage liver disease (ESLD), and finally death [2]. Various factors can shorten or accelerate this process of development (e.g., HIV infection) [3]. Likewise, some patients may develop acute-on-chronic liver failure (ACLF).

ACLF is an entity that has been defined differently in the West and East. Asian Pacific Association for the Study of Liver (APASL) defines ACLF as “acute hepatic insult manifesting as jaundice and coagulopathy complicated within 4 weeks by clinical ascites and/or encephalopathy in a patient with previously diagnosed or undiagnosed chronic liver disease/cirrhosis, and is associated with a high 28-day mortality” [4]. On the other hand, European association for the study of liver (EASL) and American association for the study of liver diseases (AASLD) define ACLF as “acute deterioration of pre-existing chronic liver disease usually related to a precipitating event and associated with increased mortality at 3 months due to multi-system organ failure” [5]. Moreover, others definitions have been proposed in order to establish a consensus [6]. Although there is no consistent definition of ACLF, all of them consider the hepatitis E virus infection as a precipitating event.

In this review, the role of acute hepatitis E virus (HEV) infection on ACLF was analyzed, focusing on the impact of this infection on patient survival and prognosis in several world regions.

## 2. Main Text

### 2.1. Impact of Acute HEV Infection on CLD in Asia

Asia is an HEV-endemic area and several outbreaks have been reported [7]. The four major HEV genotypes have been reported in this area [8]. Nevertheless, the majority of cases in India and China are due to HEV genotype 4, whereas the majority of cases in Japan are due to genotypes 3 and 4 [9]. The first report linking HEV infection to liver decompensation was in Pakistan [10]. In that study, 233 CLD patients were tested for hepatitis A (HAV) and HEV antibodies. Interestingly, the cause of sudden liver decompensation in all 4 patients with ACLF included in the study (100%) was HEV superinfection. Seroprevalence of HAV was considerably higher than the seroprevalence of HEV (97.8% and 17.5%), probably because 97% to 100% of patients with CLD in this highly endemic area have been immunized against HAV infection (the HAV vaccine has been available since 1999) [11]. In contrast, the rate of HEV immunization in patients is much lower. Accordingly, HEV infection could be an important trigger of liver decompensation in CLD patients in this area. An increasing number of studies in this setting have evaluated this association in HEV-endemic areas in Asia (summarized in**Table 1**).

The majority of studies have been conducted in India. The largest study was performed by Acharya et al. in a cohort of 107 cirrhotic patients [12]. Patients included in this study were classified into three groups: (i) cirrhotic patients with ACLF (n = 42), (ii) cirrhotic patients with previous CHD (n = 32), and (iii) stable cirrhotic patients with a Child-Turcotte-Pugh (CTP) score < 6 (n = 33). Thirty patients had detectable serum HEV-RNA at inclusion. The distribution of HEV-infected patients according to the three different study groups was as follows: 50% in Group 1 (21 of 42), 19% in Group 2 (6 of 32), and 10% in Group 3 (3 of 33). Consequently, in this study, the cause of liver failure in 50% of patients with ACLF was HEV infection. Of these 21 patients, mortality at week 4 of follow-up was 61.9% (n = 13), with 1-year mortality of 100%. By multivariate analysis, HEV infection was identified as a risk factor associated with mortality in ACLF (RR = 1.88), together with other clinical parameters, such as Child-Pugh score, renal failure, and sepsis. Another study performed in northern India found that the cause of ACLF in 66.1% of 121 patients was HEV infection [13]. In that study, 3-month mortality among HEV-infected patients was 43.8%. A study performed by Garg et al. analyzed 91 patients with a first ACLF and no previous history of liver decompensation. The cause of the insult to the liver was identified as HEV infection in 14 patients (15.3%) [14]. Kumar et al. identified a series of 48 patients with ACLF and HEV infection was noted in 14.5% of patients [15]. In another study performed in Delhi that included 42 patients with acute exacerbation of chronic hepatitis B (HBV) infection, HEV infection was detected in 18.6% [16]. Other studies enrolling smaller samples of patients identified liver failure as being due to HEV infection in 100% of CLD patients [17, 18]. In other studies, the percentage of liver decompensation due to HEV infection in India was considerably lower than that reported in previously studies. The study by Das et al. included patients with chronic HBV infection with liver decompensation, and HEV infection was identified as the cause of liver failure in 8.3% of patients (6 out of 72) [19]. In another study with 52 patients, the rate of ACLF caused by acute HEV infection was 9.6% [20]. In a study performed by Museja et al. in Chandigarh (a city in northern India), the cause of acute deterioration of liver function was identified as HEV in only 8 (8%) of 100 patients included with ACLF [21]. In another study conducted by the same authors, only 3.9% of patients with ACLF (4 of 102) were HEV-infected at liver failure [22]. Differences between studies could be related to the year/months when the study was performed, a concomitant HEV outbreak at the same time as the study, or HEV subtype. Nevertheless, strong evidence suggests that HEV is a major cause of liver failure in patients diagnosed with CLD in India, with an overall rate of 27.3% (Table 1). Finally, there are two studies of pediatric patients carried out in India. The first included 36 children with acute-on-chronic liver disease (ACLD), 17 of whom fulfilled ACLF criteria, and HEV infection was identified in 63.8% of these patients [23]. In the second, the presence of ACLF due to HEV infection was confirmed in 3 of 31 patients included (9.6%) [24].

Three studies conducted in South China should be highlighted. First, Zhang et al. conducted a study whose main objective was to evaluate the impact of hepatitis A virus (HAV) superinfection or HEV superinfection on patients with chronic HBV infection [25]. A total of 188 patients were included: 52 (27.6%) with HAV superinfection and 136 (72.4%) with HEV superinfection. The rate of liver failure observed was higher among HBV patients with HEV (39.7%) than with HAV (11.5%) (p = 0.002). Furthermore, the mortality rate for patients with HEV was also higher than in those infected with HAV (33.8% versus 1.9%; p < 0.001). Second, Ke et al. performed a study whose main objective was to determine the etiology of fatal liver failure in patients with chronic HBV infection. Of the 107 patients analyzed, HEV infection was identified as the cause of liver insult in 80 patients (74.7%) [26]. Lastly, another study by Zhang et al. explored the risk factors for adverse clinical outcomes in acute HEV infection. Of 512 patients with acute HEV infection included in the study, 41.2% had the infection without underlying CLD. However, 58.8% had acute-on-chronic liver disease and the liver disease-related mortality rate was 11.3% [27].

Several studies with small samples of patients have analyzed the impact of HEV infection on CLD in other Asian countries. In Bangladesh, a study performed by Mathab et al. identified HEV infection as the cause in 21.7% of 69 ACLF patients included [28]. Similarly, 14 of 32 cirrhotic patients with liver decompensation (43.75%) included in another study performed in Bangladesh had anti-HEV IgM antibodies [29]. In a study carried out in Vietnam of 382 cirrhotic patients due to HBV, an association was found between HEV infection and a higher Child-Pugh score [30]. Interestingly, the viral strain isolated in that study was consistent with HEV genotype 3. Two series of cases reported in Nepal revealed 100% HEV infection in 19 patients with ACLF [31, 32]. Finally, four cases of ACLF caused by HEV infection have been reported in Pakistan [10].

### 2.2. Impact of Acute HEV Infection on CLD in Europe

Previously, HEV infection in Europe was associated with travelers or imported cases [33]. An increasing number of studies have demonstrated that while Europe is not home to HEV outbreaks like those found in Asia or Central America, the prevalence and numbers of cases of HEV infection are steadily increasing [34, 35]. The main route of transmission in European countries is via the consumption of raw or undercooked meat (mainly pork). While the prevalence of HEV infection has been widely studied in both the general and special populations (such as HIV-infected patients and transplant recipients) [36, 37], the impact of infection on CLD has not so far been well studied in this region in contrast with Asian countries.

The first cases of liver decompensation in cirrhotic patients in Europe due to autochthonous HEV infection were documented in the United Kingdom in 2005-2006 [38]. In that study, 3 CLD patients developed liver failure due to HEV genotype 3 infection; 2 of the patients died (both cirrhotic) and 1 developed encephalopathy but recovered. Another case described in the same cohort involved a 59-year-old man with undiagnosed liver cirrhosis who acquired HEV genotype 3 infection [39]. The patient developed subacute liver failure with grade 1-2 encephalopathy and died due to infection. Subsequent data in another British study have been reported, including further cases of acute HEV infection, although no cases of acute liver failure or death have been reported in CLD patients [40]. In France, 7 cases (6 with a known history of CLD) of acute hepatitis, all HEV genotype 3, with encephalopathy have been reported [41]. Fulminant acute hepatitis was the cause of death in all but two cases. When the risk factors associated with the severe form of acute HEV were compared with a control cohort with a milder form of acute HEV infection, only concomitant CLD was identified. In another study, ninety-three cases of acute hepatitis E acquired in Switzerland have been documented. Four of these patients had experienced previous episodes of decompensation. Two of them had liver failure with a fatal outcome [42]. The etiology of decompensation was analyzed in a British/French cohort including 343 patients with decompensated chronic liver disease [43]. HEV infection was the trigger insult in 11 patients (3.2%), 3 (27%) of whom died in follow-up due to the infection. In terms of mortality however HEV infection was not statistically significant among patients who developed liver decompensation due to other causes. Another French study evaluated the impact of HEV infection on mortality rate in patients with acute alcoholic hepatitis [44]. The overall mortality rate in the 84 patients included was 28.6% and did not vary between patients with non-HEV infection (28.3%), past HEV infection (29%), and active HEV infection (33%). Finally, a longitudinal prospective study including HIV-infected patients conducted in Spain analyzed the impact of HEV acute infection on cirrhotic patients [45]. In that study, 83 patients diagnosed with liver cirrhosis were followed up for 1 year. During the study period, 8 (9.6%) patients experienced HEV infection. The presence of liver decompensation was more common in the HEV-infected patients (2/8; 25%) than in the uninfected patients (2/75; 2.6%) (p = 0.023).

Although the number of studies evaluating this association is limited in Europe, two studies have indirectly evaluated it. In 1985, Nanji and French reported that there was a correlation between national pork consumption (including 17 developed countries) and mortality from CLD [46]. These data were reevaluated and confirmed in 2010 by Dalton et al. [47], who evaluated factors associated with CLD mortality in 18 developed countries (including 14 European countries). They found that consumption of pig meat was an independent risk factor for CLD mortality, which also correlated with alcohol intake. There are several hypotheses for this association, although HEV transmission should also be taken into consideration.

### 2.3. Impact of Acute HEV Infection in CLD in Africa

In Africa, two studies have analyzed the impact of HEV infection on liver failure in CLD patients. Both studies have been based on APASL definition for ACLF. The first study was conducted in Egypt and included 100 patients with ACLF and 80% of survival rates [48]. Of these, HEV-RNA was detected in 13 (13%) patients. These patients were cirrhotic, diagnosed with hepatocellular carcinoma, and/or awaiting liver transplant with jaundice. In the second study, performed in Gambia, 40 ACLF patients were included and compared with 71 compensated cirrhotic patients [49]. Of the ACLF patients, 100% presented ascites, 25% encephalopathy, and 27.5% gastrointestinal bleeding. HEV infection was not detected in both groups. In that study, therefore, HEV infection was not related to ACLF.

### 2.4. Impact of Acute HEV Infection in CLD in America

Two studies and one case report have been reported in the USA [50]. The first study evaluated 115 patients with chronic HCV infection diagnosed with cancer (cirrhosis: n = 47). Anti-HEV IgM was not detected in any patient. In the second study by Fontana et al., only 3 patients (0.4%) in a cohort of 681 ACLF patients exhibited anti-HEV IgM [51]. During follow-up (8 serum samples tested), HEV-RNA was not amplified in any of the patients. Finally, the case report documented fatal hepatic decompensation due to HEV genotype 4 infection in a transplant recipient [52]. The patient probably acquired the infection in Hong Kong during a 7-week stay there and died with chronic HEV infection following accelerated progression of cirrhosis.

## 3. Conclusion and Perspectives

Globally, HEV infection negatively impacts the survival and prognosis of patients with CLD. This association is more apparent in Asia than in European, African, or American countries. This difference could be mainly related to the presence of viral genotypes/subtypes in each zone. The impact of HEV genotype 3 on the prognosis and outcome of CLD is controversial. In Europe, where genotype 3 is endemic, the number of reported cases of liver decompensation due to HEV infection is low (n = 23; rate of liver decompensation = 5.7%). In European countries, the impact of HEV infection on the prognosis and survival of CLD patients seems to be limited (n = 3; liver decompensation rate = 0.4%). Despite these observations, clinicians should consider HEV infection in European and American patients with CLD with a sudden deterioration of liver function, independently of their travel history.

In China, a hepatitis E vaccine has recently demonstrated high short-term and long-term protective efficacy [53, 54]. Vaccination could therefore significantly reduce transmission of this disease and so improve the prognosis of CLD patients in or from endemic areas. This vaccine has not yet been tested in certain populations, including pregnant women and the immunosuppressed cases. Consequently, the World Health Organization (WHO) does not recommend general vaccination at this time, and universal vaccination is considered exclusively during hepatitis outbreaks [55]. Nevertheless, the recommendations for vaccination in CLD patients in this setting may be revised following suggestions by several experts because of the overall high rate of decompensation due to HEV infection (33.9%) observed in studies conducted in these areas.

## Figures and Tables

**Table 1:** Studies that evaluated the impact of hepatitis E virus (HEV) infection on chronic liver disease (CLD) or acute-on-chronic liver failure (ACLF) patients.

Country	Region/City	Population	Number of patients	Cases of decompensation due to HEV infection, n (%)	Reference
ASIA					

India	New Delhi	Cirrhotic patients with liver decompensation	CHD = 31 ACLF = 42	CHD = 6 (19.3%) ACLF = 21 (50%)	[12]

India	Lucknow	ACLF patients	121	80 (66.1%)	[13]

India	New Delhi	ACLF patients	91	14 (15.3%)	[14]

India	New Delhi	ACLF patients	48	7 (14.5%)	[15]

India	New Delhi	CHBV patients with acute hepatitis	43	8 (18.6%)^1^	[16]

India	New Delhi	Cirrhotic patients with liver decompensation	10	10 (100%)	[17]

India	Vellore	ACLF patients	9	9 (100%)	[18]

India	New Delhi	CHBV patients with liver decompensation	72	6 (8.3%)	[19]

India	Jaipur	ACLF patients	52	5 (9.6%)	[20]

India	Chandigarh	ACLF patients	100	8 (8%)	[21]

India	Chandigarh	ACLF patients	102	4 (3.9%)	[22]

India	Lucknow	ACLF pediatric patients	36	23 (63.8%)	[23]

India	Chandigarh	ACLF patients	31	3 (9.6%)	[24]

China	Guangzhou	CHBV infected with HEV infection	136	54 (39.7%)	[25]

China	Guangzhou	ACLF patients	107	80 (74.7%)	[26]

China	Shanghai	ACLF patients	301	34 (11.3%)	[27]

Bangladesh	Dhaka	ACLF patients	69	15 (21.7%)	[28]

Bangladesh	Dhaka	Cirrhotic children with liver decompensation	32	14 (43.75%)	[29]

Nepal	Kathmandu	Cirrhotic patients with liver decompensation	12	12 (100%)	[31]

Nepal	Kathmandu	ACLF patients	7	7 (100%)	[32]

Pakistan	Karachi	ACLF patients	4	4 (100%)	[10]

EUROPE					

UK	London	Acute HEV infected patients	40	3 (7.5%)	[38–40]

France	Toulouse	Fulminant hepatitis failure	7	7 (100%)	[41]

Switzerland	Lausanne	Cirrhotic patients with liver decompensation	4	2 (50%)	[42]

UK/ France	Truro, Glasgow, Norwich, Toulouse	Cirrhotic patients with liver decompensation	343	11 (3.2%)	[43]

France	Villejuif	Acute alcoholic hepatitis	84	3 (3.5%)	[44]

Spain	Córdoba	HIV-infected cirrhotic patients with acute HEV infection	83	2 (2.4%)	[45]

AFRICA					

Egypt	Mansoura	ACLF patients	100	13 (13%)	[48]

Gambia	Banjul	ACLF patients	40	0	[49]

AMERICA					

USA	Houston	HCV cirrhotic patients with cancer	47	0	[50]

USA	Ann Arbor, Bethesda, Maywood, Philadelphia, Pittsburgh, Charleston, Dallas	ACLF patients	681	3 (0.4%)	[51]

Number of cases (n); hepatitis E virus (HEV); chronic hepatic decompensation (CHD); acute-on-chronic liver failure (ACLF); hepatitis A virus (HAV); chronic hepatitis B virus (CHBV); United Kingdom (UK); human immunodeficiency virus (HIV). ^1^  Two cases were superinfected with both HAV and HEV.
